# Cancer Attributable to Tobacco Smoking in Member Countries of Association of Southeast Asian Nations (ASEAN) in Year 2018

**DOI:** 10.31557/APJCP.2019.20.10.2909

**Published:** 2019

**Authors:** Susi Ari Kristina, Ni Putu Ayu Linda Permitasari, Kadek Ida Krisnadewi, Karina Anindita Santoso, Pia Rika Puspawati, Wa Ode Masrida, Yuni Andriani

**Affiliations:** 1 *Department of Pharmaceutics, *; 2 *Graduate student in Pharmacy Management, Faculty of Pharmacy, Universitas Gadjah Mada, Indonesia.*

**Keywords:** Burden of disease, tobacco smoking, cancer, smoking attributable fraction, ASEAN

## Abstract

South East Asia is one of the world’s largest tobacco epidemic regions which tobacco smoking is known increase the risk of various diseases, including cancer. As data from GLOBOCAN 2018 has had released on September 2018, the aim of this study are to calculate the estimated burden of several types of cancer attribu to tobacco smoking in Association of Southeast Asian Nations (ASEAN) 2018 and compare it with established result data in 2012. So it can be highlight what has been achieved and what it needs to be addressed by member countries of ASEAN to strengthen cancer prevention against tobacco smoking. This study was using descriptive epidemiological incidence and prevalence-based research design to estimate the burden of 14 types of cancer attributable to tobacco smoking in member countries of ASEAN, in term of incidence and mortality. The cancer incidence and mortality data gained from GLOBOCAN 2018. According to the estimation, tobacco smoking was responsible for 121,849 new cancer cases in 2018 (106,858 male and 14,991 female cases) in ASEAN 2018. Our findings are mostly lower than previous study in 2012, both for cancer incidence and mortality in male and female. It seems more ASEAN member states are adopting effective policies in the MPOWER suite of interventions such as raising taxes on tobacco, establishing smoke-free areas and implementing graphic health warnings in decreasing number of tobacco smoking. Therefore, ASEAN member countries are strongly encouraged to strengthen the existing tobacco control measure in order to effectively gain a significant decline of tobacco smoking related cancer in the future.

## Introduction

There are more than one billion tobacco users worldwide and 80% of them live in low and middle income countries. As per estimates, 246 million smokers and 290 million smokeless tobacco users in the World Health Organization (WHO) South East Asia Region which makes it one of the largest tobacco consuming regions (World Health Organisation South-East Asia Regional Office (WHO SEARO), 2017). Those numbers are still become a global health problem, because both smoking and smokeless tobacco users have well known increase the risk of death from various diseases such as coronary heart disease, stroke, and respiratory disorders in adults, and many types of cancer which may increase the health burden of nations (World Health Organization (WHO), 2011a)

Smoking increases the risk of malignant tumors in nearly all organs with at least 30% of all cancer deaths smoking is attributed to smoking (Bassiony et al., 2015). Once tobacco has damaged cells, they can grow uncontrollably as cancer. When DNA is damaged, a cell can begin growing out of control and create a cancer tumor. This happens because poisons in tobacco smoke can destroy or change the cell’s instructions that might damage DNA in a way that leads to cancer (US Department of Health and Human Services, 2010). The long time lags for development of cancer associated with smoking, the impact of smoking caused diseases on mortality in many regions estimates will continue to rise for at least two decades (World Health Organization (WHO), 2009).

At present, there are 121 million adult smokers, approximately 10% of world’s smoker, in member countries of Association of Southeast Asian Nations (ASEAN) countries (Kimman et al., 2015). ASEAN was established on 8 August 1967 and consists of eleven countries namely Brunei Darussalam, Cambodia, Indonesia, Lao PDR, Malaysia, Myanmar, Philippines, Singapore, Thailand, Vietnam, and Timor Leste in South East Asia region. The member countries have a combined population of approximately 625 million people, accounted for 9% of the world’s population (Kristina et al., 2016).

South East Asia is one of the world’s largest tobacco epidemic regions (Katanoda et al., 2014). Based on Kristina et al., (2016) study, tobacco smoking was responsible for about 131,502 cancer cases (28.4%) and 105,830 cancer deaths (30.5%) in ASEAN 2012 using data from GLOBOCAN 2012. As data from GLOBOCAN 2018 has had released on 12 September 2018 (World Health Organisation (WHO) IARC, 2018), the aim of this study to calculate the estimated burden of several types of cancer due to smoking in ASEAN 2018 and compare it with result data in 2012. So through this study, it can be highlight what has been achieved and what it needs to be addressed by member countries of ASEAN to strengthen cancer prevention against tobacco smoking.

## Materials and Methods

This research was using descriptive epidemiological incidence and prevalence-based research design to estimate the burden of cancer due to smoking in members of ASEAN. There were three steps to gain the data; first we selected smoking-related cancer and estimated population-related smoking attributable fraction (SAF) using relative risks and prevalence of tobacco smokers in each member countries of ASEAN. Second, we find the number of incidence and cancer mortality of each country. Then lastly, we estimated smoking attributable incidence and mortality from the number of incidence and mortality multiply by SAF. 

*Smoking prevalence*

The prevalence of current tobacco smokers among individuals aged 15 years and older, published by WHO global report on trends in prevalence of tobacco smoking 2000-2001, was obtained from Kristina et al., (2016) study (Kristina SA et al., 2016). As the burden of cancer observed in 2018 reflects the past exposure of smoking, the prevalence in the year 2000 was used in our study due to long time lags for development of cancer associated with smoking (World Health Organization (WHO), 2009). Timor Leste country prevalence of smoking was not included since the year of 2000’s data was not available. As shown in Table 1, the highest prevalence of tobacco smoking both among male was in Lao PDR (71.5%), followed by Philippines (59.1%), and Malaysia (56.0%). Meanwhile the highest prevalence of tobacco smoking among female in ASEAN member countries found in Lao PDR (19.9%), Myanmar (14.7%), and the Philippines (11.0%), respectively. Generally, the prevalence of smoker was quite lower in female than in male. 

**Table 1:** Prevalence of Tobacco Smoking among ASEAN Countries

Country/year	Number of population (million)	Prevalence of Tobacco smoking	
		Male (%)	Female (%)
Brunei/2,000	0.4	29.4	4.8
Cambodia/2,000	14.5	44.5	7.1
Indonesia/2,000	237.7	54.8	4.9
Lao PDR/2,000	6.4	71.5	19.9
Malaysia/2,000	28.9	56	3
Myanmar/2,000	60.4	55.2	14.7
Philippines/2,000	95.8	59.1	11
Singapore/2,001	3.8	24.2	3.5
Thailand/2,000	67.6	46.8	2.9
Viet Nam/2,000	87.8	49.4	1.6

Source, Kristina et al., (2016)

**Table 2:** Relative Risks and Smoking Attributable Fractions (SAFs) of Selected Cancer in ASEAN Countries

Gender	Cancer	RR	SAF									
			Bru	Cam	Ind	Lao	Mal	Mya	Phi	Sing	Tha	Vie
Male	Lip, oral cavity	3.43	0.42	0.52	0.57	0.63	0.58	0.57	0.59	0.37	0.53	0.54
	Nasopharynx	1.95	0.22	0.3	0.34	0.4	0.35	0.34	0.36	0.19	0.31	0.31
	Pharynx	6.76	0.63	0.72	0.76	0.8	0.76	0.76	0.77	0.58	0.73	0.74
	Esophagus	2.5	0.31	0.4	0.45	0.52	0.46	0.45	0.47	0.27	0.41	0.42
	Stomach	1.74	0.18	0.25	0.29	0.35	0.29	0.29	0.3	0.15	0.26	0.26
	Colorectal	1.13	0.04	0.05	0.07	0.09	0.07	0.07	0.07	0.03	0.06	0.06
	Liver	1.56	0.14	0.2	0.23	0.29	0.24	0.24	0.25	0.12	0.21	0.21
	Pancreas	1.7	0.17	0.24	0.28	0.33	0.28	0.28	0.29	0.14	0.25	0.25
	Larynx	6.98	0.64	0.73	0.77	0.81	0.77	0.77	0.78	0.59	0.74	0.74
	Lung	8.96	0.7	0.78	0.81	0.85	0.82	0.81	0.82	0.66	0.79	0.79
	Cervix uteri	NA	NA	NA	NA	NA	NA	NA	NA	NA	NA	NA
	Ovary	NA	NA	NA	NA	NA	NA	NA	NA	NA	NA	NA
	Kidney	1.52	0.13	0.19	0.22	0.27	0.23	0.22	0.24	0.11	0.2	0.2
	Bladder	2.77	0.34	0.44	0.49	0.56	0.5	0.49	0.51	0.3	0.45	0.46
Female	Lip, oral cavity	3.43	0.1	0.15	0.11	0.33	0.07	0.26	0.21	0.08	0.07	0.04
	Nasopharynx	1.95	0.04	0.06	0.04	0.16	0.03	0.12	0.09	0.03	0.03	0.01
	Pharynx	6.76	0.22	0.29	0.22	0.53	0.15	0.46	0.39	0.17	0.14	0.08
	Esophagus	2.5	0.07	0.1	0.07	0.23	0.04	0.18	0.14	0.05	0.04	0.02
	Stomach	1.45	0.02	0.03	0.02	0.08	0.01	0.06	0.05	0.02	0.01	0.01
	Colorectal	1.4	0.02	0.03	0.02	0.07	0.01	0.06	0.04	0.01	0.01	0.01
	Liver	1.56	0.03	0.04	0.03	0.1	0.02	0.08	0.06	0.02	0.02	0.01
	Pancreas	1.7	0.03	0.05	0.03	0.12	0.02	0.09	0.07	0.02	0.02	0.01
	Larynx	6.98	0.22	0.3	0.23	0.54	0.15	0.47	0.4	0.17	0.15	0.09
	Lung	8.96	0.28	0.36	0.28	0.61	0.19	0.54	0.47	0.22	0.19	0.11
	Cervix uteri	1.83	0.04	0.06	0.04	0.14	0.02	0.11	0.08	0.03	0.02	0.01
	Ovary	2.07	0.05	0.07	0.05	0.18	0.03	0.14	0.11	0.04	0.03	0.02
	Kidney	1.52	0.02	0.04	0.02	0.09	0.02	0.07	0.05	0.02	0.01	0.01
	Bladder	2.77	0.08	0.11	0.08	0.26	0.05	0.21	0.16	0.06	0.05	0.03

**Table 3:** Smoking Attributable Cancer Incidence in ASEAN Countries, 2018

Gender	Cancer	Smoking attributable cancer incidence, 2018										Total
		Bru	Cam	Ind	Lao	Mal	Mya	Phi	Sin	Tha	Vie	
Male	Lip, oral cavity	0	90	1,656	52	148	843	409	41	1,095	639	4,973
	Nasopharynx	6	48	4,520	54	535	470	633	75	437	1,309	8,087
	Pharynx	0	78	743	47	98	1,207	308	47	894	1,487	4,908
	Esophagus	1	76	339	92	111	1,147	314	38	1,007	775	3,901
	Stomach	3	50	523	109	189	1,031	404	37	396	2,403	5,146
	Colorectal	3	29	1,099	23	176	154	512	42	385	370	2,792
	Liver	4	282	2,796	172	289	754	1,424	81	2,752	3,810	12,363
	Pancreas	1	14	644	9	118	96	370	50	240	113	1,657
	Larynx	1	69	2,056	41	286	798	792	62	771	959	5,835
	Lung	27	618	15,599	455	2,339	3,030	8,049	909	8,098	10,891	50,016
	Cervix uteri	NA	NA	NA	NA	NA	NA	NA	NA	NA	NA	0
	Ovary	NA	NA	NA	NA	NA	NA	NA	NA	NA	NA	0
	Kidney	2	12	283	8	103	57	282	74	142	203	1165
	Bladder	2	51	2,327	29	230	1,757	405	72	737	404	6014
	Total	50	1,419	32,585	1,092	4,621	11,346	13,902	1,528	16,953	23,363	106,858
Female	Lip, oral cavity	0	22	188	19	18	158	100	4	78	16	603
	Nasopharynx	1	5	167	13	13	78	76	5	15	21	393
	Pharynx	0	10	94	5	8	73	42	3	26	32	293
	Esophagus	0	5	15	3	4	127	22	1	16	5	197
	Stomach	0	5	13	15	7	140	44	4	18	34	279
	Colorectal	1	15	171	21	25	102	220	13	71	35	674
	Liver	0	25	83	26	6	118	110	3	82	41	493
	Pancreas	0	2	48	3	6	24	74	5	15	4	180
	Larynx	0	3	44	4	6	41	54	1	15	17	185
	Lung	10	168	1,581	131	188	1,491	1,805	124	1,009	568	7,076
	Cervix uteri	2	51	1,201	43	38	680	564	8	183	50	2,819
	Ovary	2	25	603	27	37	214	482	13	86	23	1,513
	Kidney	0	1	11	2	4	9	30	2	5	8	71
	Bladder	0	4	75	3	7	44	50	4	21	6	214
	Total	17	341	4,293	313	367	3,299	3,673	189	1,640	860	14,991
Total		67	1,759	36,877	1,405	4,988	14,644	17,576	1,717	18,593	24,223	121,849

**Table 4:** Smoking Attributable Cancer Mortality ASEAN Countries, 2018

Gender	Cancer	Smoking attributable cancer mortality, 2018										Total
		Bru	Cam	Ind	Lao	Mal	Mya	Phi	Sin	Tha	Vie	
Male	Lip, oral cavity	0	56	761	29	68	480	193	18	571	288	2,463
	Nasopharynx	3	39	2,839	40	298	350	402	47	293	857	5,167
	Pharynx	0	53	379	27	48	781	158	23	478	778	2,725
	Esophagus	0	76	297	86	100	1,125	286	34	894	689	3,586
	Stomach	2	40	399	92	136	821	296	32	347	1,854	4,,020
	Colorectal	1	17	530	13	79	88	239	20	208	169	1,365
	Liver	3	284	2,757	172	286	764	1,408	72	2,770	3,847	12,363
	Pancreas	1	15	630	9	113	93	363	42	252	102	1,620
	Larynx	0	39	922	22	127	440	366	18	433	425	2,792
	Lung	18	599	13,456	438	1,970	2,997	7,204	819	8,037	9,359	44,896
	Cervix uteri	NA	NA	NA	NA	NA	NA	NA	NA	NA	NA	0
	Ovary	NA	NA	NA	NA	NA	NA	NA	NA	NA	NA	0
	Kidney	1	9	153	5	44	33	129	16	82	105	574
	Bladder	0	26	955	12	85	126	144	18	372	163	1,902
	Total	29	1,251	24,077	945	3,354	8,097	11,188	1,159	14,735	18,637	83,472
Female	Lip, oral cavity	0	12	74	9	7	80	41	2	39	6	271
	Nasopharynx	0	3	89	8	7	52	41	2	8	13	224
	Pharynx	0	6	38	3	3	46	17	1	12	17	143
	Esophagus	0	5	12	3	4	116	19	1	13	4	176
	Stomach	0	4	10	13	5	113	33	2	16	26	222
	Colorectal	0	9	81	11	11	58	101	6	37	16	331
	Liver	0	25	80	25	5	119	107	3	80	41	486
	Pancreas	0	2	46	2	5	23	70	5	12	4	170
	Larynx	0	2	25	3	3	29	33	0	12	10	117
	Lung	7	162	1,283	124	148	1,440	1,473	110	989	462	6,197
	Cervix uteri	1	34	628	22	19	387	292	4	103	25	1,516
	Ovary	1	15	329	15	22	130	269	8	51	13	853
	Kidney	0	1	6	1	2	5	13	1	3	4	35
	Bladder	0	3	35	1	3	24	19	1	10	2	98
	Total	9	284	2,737	242	244	2,622	2,528	146	1386	642	10,840
Total		38	1,535	26,815	1,187	3,599	10,718	13,716	1,305	16,121	19,279	94,312

*Selection of smoking-related cancer*

According to the level of evidences, we decided to include fourteen types of cancer with positive association with tobacco smoking shown by relative risk (RR) value more than one in this analysis, provided by Kristina et al., (2016) study. The cancer included cancer of lip and oral cavity (C00-08), nasopharynx (C11), others pharynx (C09–10,C12–14), esophagus (C15), stomach (C16), colorectal (C18–20), liver (C22), pancreas (C25), larynx (C32), lung cancer (C33–34), cervix uteri (C53), ovary (C56), kidney (C64–66), and urinary bladder (C67). Most RR of tobacco smoking-related cancer were in that study derived from recent meta-analysis (Gandini et al., 2008), which consisted of 254 reports, of which 8 were from Africa, 28 were from Japan, 8 were from India, 39 were from China, 13 were from South America, and the others were from western countries (Kristina et al., 2016). That global meta-analysis was selected because it is the most updated and there is no many meta-analysis conducted in Asia exists.

*Incidence and cancer mortality*

The number of cancer incidence and mortality in ASEAN member countries in 2018 were derived from GLOBOCAN 2018. GLOBOCAN is an online database from WHO International Agency for Research on Cancer (IARC) that providing estimates of incidence and mortality in 185 countries for 36 types of cancer (International Agency for Research on Cancer (IARC), 2012). The methods of estimation are country specific and the quality of the estimation depends upon the quality and on the amount of the information available for each country. Incidence data are derived from national cancer registries (Bray et al., 2018). If country data is not available, estimation by modeling, using incidence mortality ratios derived from recorded data in country or local cancer registries is used. Mortality statistics were based on national data that are collated and made available by the WHO for countries with vital registration (Bray et al., 2018). Incidence and mortality from ten countries in Southeast Asia region were available, while Timor Leste data was not available yet for incidence and mortality of cancers in 2018. 

*Estimation of smoking attributable fraction (SAF) of cancer*

To calculate SAF values due to cancer, two parameters were considered: 1) the relative risks of tobacco smoking for the different related cancer, and 2) prevalence of smokers for male and female. The formula of SAF is shown below,

${\mathrm{SAF}}_{s}=\frac{p(\mathrm{PRi}-1)}{1+p(\mathrm{rri}-1)}$

where “p” is the prevalence of smokers in the national population, “RR” is the relative risk of illness due to tobacco smoking, sub-script-i is a category of disease. Exception for Singapore, due to there has been no any prevalence data of tobacco smoking, the SAF value gained and used directly from prevalence-based study of Gandini et al., (2008). 

## Results

Relative risks and SAF of countries members of ASEAN reported in Table 2. As the strongest association with smoking was found for lung cancer (World Health Organization (WHO), 2011b), tobacco smoking is attributable to about 3% - 89.6% for male population in ASEAN member countries. Meanwhile for female, tobacco smoking is attributable to lung cancer about 11% - 89.6%. 

Furthermore, smoking attributable cancer incidence in ASEAN member countries has shown in Table 3. According to the estimation, tobacco smoking accounted for 121,849 new cancer cases in 2018 (106,858 male and 14,991 female cases). In male, the total number of tobacco smoking attributable cancer incidence was the highest in Indonesia (32,585), followed by Viet Nam (23,363 cases), and Thailand (16,953 cases). On the other hand, the three highest number of tobacco smoking attributable cancer incidence in female was found in Indonesia (4,293 cases), Philippines (3,673) and Myanmar (3,299 cases). In male, the number of patients with lung cancer (50,016) attributable to tobacco smoking was the highest followed by liver cancer (12,363) and nasopharynx cancer (8,087). Meanwhile, in female, the number of patients with lung cancer (7,076) attributable to tobacco smoking was also the highest followed by cervix uteri cancer (2,819) and ovary cancer (1,513). 

On the other hand, total cancer mortality in ASEAN due to tobacco smoking accounted for 94,312 which consisted of 83,472 and 10,840 cancer mortality for male and female respectively as shown in Table 4. Same as cancer incidence in male, the total number of smoking attributable cancer mortality was the highest in Indonesia (24,077), followed by Viet Nam (18,637), and Thailand (14,735). On the other hand, the three highest number of smoking attributable cancer mortality in female was found in Indonesia (2,737), Myanmar (2,622), and Philippines (2,528). In male, the number of patients with lung cancer mortality attributable to tobacco smoking was the highest (44,896) followed by liver cancer (12,363), and nasopharynx (5,167). Same as in male, for female, the number of patients with lung cancer mortality attributable to smoking was the highest (6,197), followed by cervix uteri (1,516) and ovary (853). 

## Discussion

It is clear that cancer is an urgent global challenge that need prevention, early detection and diagnosis, treatment, and care services. Meanwhile tobacco consumption also remains a significant threat to public health and associated with different types of cancer (Bassiony et al., 2015; Islami et al., 2015). This study found that tobacco smoking was responsible for 121,849 new cancer cases in 2018 (106,858 male and 14,991 female cases) in ASEAN 2018. Our findings are slightly lower than previous study in 2012, with total of new cancer cases was 131,502 (114,775 cancer incidence in male and 16,727 in female, higher than in 2018, both for male and female (Kristina et al., 2016).

On the other hand, the comparison of cancer mortality due to tobacco smoking of male between data from 2012 and 2018 is shown in Figure 1. From the figure, it can be seen that the cancer mortality was mostly lower in 2018 than 2012 for bladder, kidney, lung, pancreas, liver, colorectal, stomach, esophagus, pharynx and lip, oral cavity cancer. Only larynx and nasopharynx cancer have had higher number of mortality in 2012 than 2018. In addition, for female, cancer mortality was also mostly lower in 2018 than 2012 in bladder, kidney, lung, larynx, pancreas, liver, colorectal, stomach, esophagus, pharynx, lip, oral cavity cancer. Only female mortality of ovary, cervix uteri and nasopharynx cancer in 2018 were higher than 2012.

Incidence data are produced by population based cancer registries. According to recent data compiled in Volume XI of the IARC’s Cancer Incidence in Five Continents, approximately 15% of the world population was covered by high-quality cancer registries around 2010, with lower registration in Asia only 6.5% of the total population (Bray et al., 2018). As well as for number of mortality, the recorded data from lower resource countries are the only relatively unbiased source of information on the distribution of common cancer types in a defined population. Lower number of cancer incidence and mortality between 2018 and 2012 in member countries of ASEAN may drive by different number cancer registries in those years. 

In addition, contemporary information on the fraction of cancer that potentially could be prevented, like tobacco smoking, is useful for priority setting in cancer prevention and control (Islami et al., 2018). For many years, WHO has worked with governments worldwide to strengthen policies, programs and strategies that prevent and reduce tobacco use, like MPOWER which measures assist in reducing the demand for tobacco products at country-level (Singh, 2012; World Health Organization (WHO), 2013). Global experiences have revealed prevalence of tobacco smoking can effectively decrease with implementation of MPOWER policies (Malhi et al., 2015 ; Heydari et al., 2016; Kumar and Purushothama, 2018). More member countries of ASEAN are showing leadership in adopting effective policies in the MPOWER suite of interventions such as raising taxes on tobacco, establishing smoke-free areas and implementing graphic health warnings (Amul and Pang, 2017).

Report tracks implementation progress of tobacco tax policy against the WHO Framework convention on tobacco Control (FCTC) in 10 ASEAN countries (WHO, 2011). Southeast Asia tobacco Control Alliance (SEATCA) believes that better health of the population as result of higher excise taxes on tobacco will lead to better labor productivity, speedier economic development and to less suffering in the region (Southeast Asia tobacco Control Alliance (SEATCA), 2017). Evidence from India and Bangladesh suggested that taxation had significantly reduced tobacco use among adults (John et al., 2018). Price policies involving taxation have been also used to control smoking not only in Thailand but also in other countries around the world. In Thailand, consumption of Tobacco products was found to have decreased (Gupta et al., 2016) relatively continuous in relation to that of other goods (World Health Organisation (WHO), 2011). Price policies through taxation to combat smoking have been very active over the period after 1996 in Thailand, consequently, Tobacco products have become relatively more expensive (Sarntisart, 2011). 

In addition, communicating the health effects of smoking using vivid, large and prominent pictorial health warnings (PHWs) on Tobacco packages remains a primary goal of national Tobacco control policy. From a public health perspective, Tobacco packaging serves as the most cost effective communications channel for governments to convey the health risks of Tobacco use, especially among those with low literacy levels. To date, more than 80 countries and territories across the globe, including ten ASEAN countries, have analyzed PHW requirements in accordance to WHO FCTC. South East Asia is only region in the world where all its countries implemented PHWs on Tobacco packages (Tan, 2016) and constantly put efforts into the Tobacco control field with high commitment from the government, scientists and activists through MPOWER implementation (Minh et al., 2016). 

Therefore, ASEAN member countries are strongly encouraged to strengthen the existing Tobacco control (Centers for Disease Control and Prevention (CDC), 2012) measure in order to effectively gain a significant decline of Tobacco smoking related cancer in the future. ASEAN member countries need to further invest and innovate on Tobacco control and promoting healthy lifestyles to achieve the target on decrease number of cancer attributable Tobacco smoking. However, the study results may underestimate the overall proportion of cancer incidence and mortality attributable to Tobacco smoking, because many others risk factors associated with cancer such as diet, body mass index (BMI), and alcohol consumption (Peppone et al., 2009; Peppone et al., 2010) are not included in the research model and thus have the potential to cause bias. Furthermore, due to substantial economic development in ASEAN might be changed dramatically so that the SAF may need to be re-estimated in order to gain more accurate estimation. 

This study found that Tobacco smoking was responsible for 121,849 new cancer cases in 2018 (106,858 male and 14,991 female cases) in ASEAN 2018. Our findings are mostly lower than previous study in 2012, both for cancer incidence and mortality in male and female. More ASEAN member states are showing leadership in adopting effective policies in the MPOWER suite of interventions such as raising taxes on Tobacco, establishing smoke-free areas and implementing graphic health warnings. Therefore, ASEAN member countries are strongly encouraged to put in place stronger Tobacco control policies and to strengthen the existing Tobacco control measure in order to effectively control cancer in the future.
